# Clinical efficacy of short-term pre-operative halo-pelvic traction in the treatment of severe spinal deformities complicated with respiratory dysfunction

**DOI:** 10.1186/s12891-020-03700-9

**Published:** 2020-10-08

**Authors:** Longtao Qi, Beiyu Xu, Chunde Li, Yu Wang

**Affiliations:** Department of Orthopaedics, Peking University First Hospital, Xicheng District, Beijing, 100034 China

**Keywords:** Severe spinal deformity, Halo-pelvic traction, Short-term, Pulmonary function

## Abstract

**Background:**

Halo traction has been used as an adjunctive method in the treatment of severe spinal deformities. But there are few reports on the clinical efficacy of halo-pelvic traction (HPT) in the treatment of severe spinal deformities complicated with respiratory dysfunction. This study was to evaluate the clinical efficacy and complications associated with pre-operative HPT in the treatment of severe spinal deformities with respiratory dysfunction.

**Methods:**

Thirty patients with severe spinal deformities complicated with respiratory dysfunction treated with short-term pre-operative HPT were retrospectively reviewed. Inclusion criteria were: (1) patients with severe kyphoscoliosis (coronal Cobb angle or kyphosis angle ≥100°) and respiratory failure, (2) patients undergoing HPT until posterior fusion surgery. All patients underwent general anesthesia for HPT application, which the pelvic ring used in this study was a half-ring, and the rods were all placed on the anterolateral side of the truck.

**Results:**

The major coronal curve scoliosis averaged 116.00 ± 16.70° and was reduced to 63.23 ± 14.00° after HPT, 46.33 ± 10.70° after surgery. The major kyphosis was 102.40 ± 27.67° and was reduced to 52.23 ± 14.16° after HPT, 42.0 ± 11.92° after surgery. A significantly increased FVC was observed after HPT (*p* < 0.001), with a significantly improved FVC% (*p* < 0.001). Similarly, a significantly increased FEV1 was also observed (*p* < 0.001), with a significantly improved FEV1% (*p* < 0.001).

**Conclusion:**

This study indicated that the modified HPT could be used to help patients with severe spinal deformities complicated with respiratory dysfunction achieve significant correction in both the coronal and sagittal deformities during the pre-operative treatment period along with improved respiratory function and in the absence of severe complications.

## Background

Severe spinal deformities are usually accompanied by cardiopulmonary impairment, and this often results in significantly increased morbidity and mortality. Despite substantial improvements in surgical techniques for the treatment of severe spinal deformities, the management of these patients remains a major challenge due to the associated poor pulmonary function, high potential for bleeding, and related neurological complications [1–3]. To minimize the risks and complications associated with these patients, especially in regard to neurological complications, numerous authors have advocated that one-stage surgery should be avoided, and adjunctive methods, such as traction (halo-femoral, halo-pelvic, halo-gravity), should be used for the reduction of stiff curves prior to surgery as well as to reduce the use of acute corrective maneuvers [3–5]. Currently, numerous surgeons advocate for halo-gravity traction (HGT) as a safe adjunctive method, as prior studies have demonstrated that pre-operative HGT can obtain 15 to 38% correction in scoliosis and 17 to 35% correction in kyphosis; in addition, some reports indicate significant improvements in pulmonary function with this procedure [3–5].

In the 1970s, O’Brien et al. introduced the application halo-pelvic traction (HPT) for patients with scoliosis in a series of 118 patients [6]. The authors reported an impressive correction of the deformities. Although the powerful traction forces generated by HPT can effectively correct various spinal deformities, the traditional HPT procedure has some drawbacks, including a number of various complications and poor tolerance due to the rods that are circularly distributed around the trunk, which tend to affect the patient’s supine sleeping position. With the development of internal fixation techniques, which do not present the drawbacks of HPT, the use of HPT has gradually declined. To the best of our knowledge, there are few reports on the clinical efficacy of HPT in the treatment of severe spinal deformities complicated with respiratory dysfunction over the last 10 years. However, our experience has led to an improved HPT technique, and the purpose of this study was to evaluate the clinical efficacy and complications associated with pre-operative HPT used for the treatment of severe spinal deformities with respiratory dysfunction.

## Methods

A total of 30 patients with severe spinal deformities complicated with respiratory dysfunction treated with pre-operative HPT were retrospectively reviewed. All of the surgeries were performed by the same senior author between 2017 and 2019. Clinical and radiographic data were collected and evaluated by an independent spinal surgeon who was not involved in the surgical treatment. Patients who met the following inclusion criteria were recruited in the study: (1) patients with severe kyphoscoliosis (coronal Cobb angle or kyphosis angle ≥100°) and respiratory dysfunction (defining as pressure of oxygen (PaO2) < 60 mmHg), and (2) patients undergoing HPT until posterior fusion surgery. Exclusion criteria were: (1) patients who had prior spinal surgery and (2) patients with revision surgery or anterior release. Informed consent was obtained from each patient prior to recruitment. The demographic data collected included age, sex, standing height, diagnosis, and duration of traction.

### Halo-Pelvic Traction (HPT) protocol

**Fig. 1:**
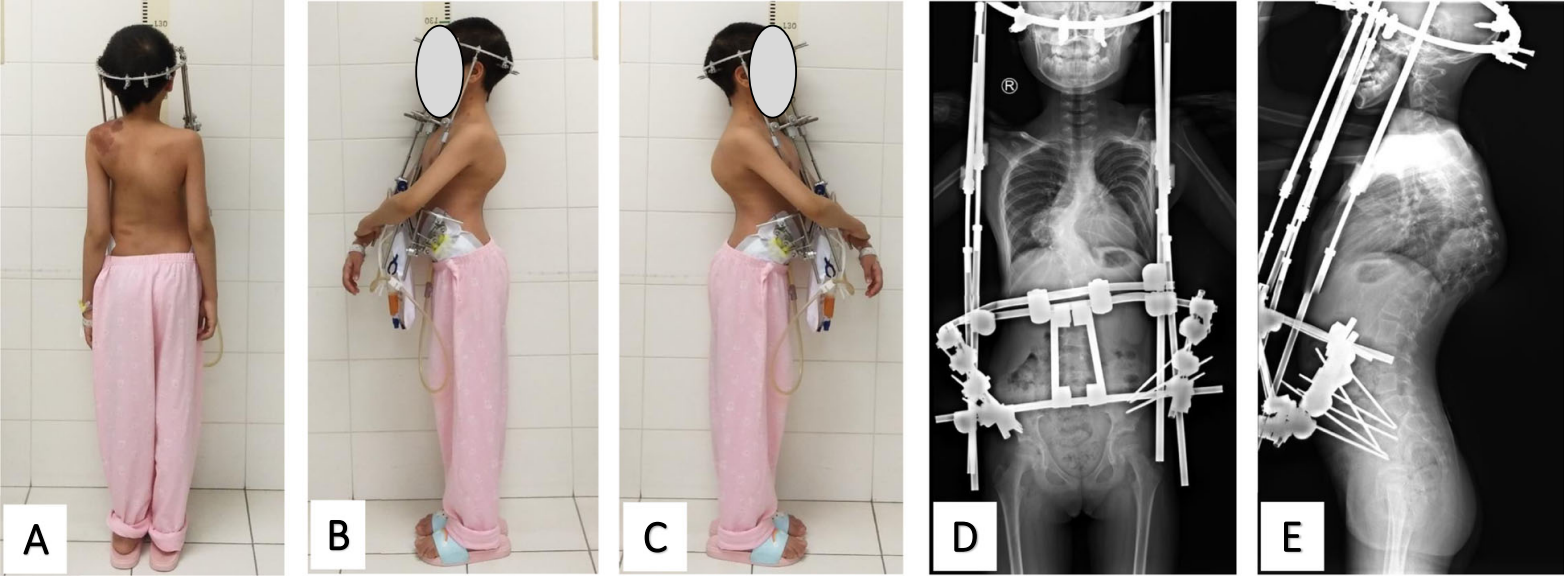
The halo-pelvic traction. The posterior (**a**) and lateral (**b/c**) views photographs, and the standing anteroposterior (**d**) and lateral digital radiographs (**e**)

The HPT device consisted of a head ring, a pelvic ring, and retractable connecting rods. The number of pins was based according to the patient’s bone condition and anatomical variation. All patients underwent local anesthesia for HPT application. Briefly, with the patient in the supine position, three pins (4.5 mm in diameter) were inserted into the area between the inner and outer table of the ilium on each side. The skull halo device was then placed using standard techniques, as previously reported [7]. Different from traditional HPT, the pelvic ring used in this study was a half-ring, and the rods were all placed on the anterolateral side of the truck (Fig. 1). The frame was constructed 3–5 days after HPT application so that the patient had time to become accustomed to the pins. Traction was initiated after the construction of the frame. For the first week, the frame was elongated at a rate of 0.5 cm per day. Beginning in the second week, the elongation rate was decreased to 0.3–0.5 cm every 2–3 days. Additionally, daily pin site care was performed. Cranial nerve and upper/lower extremity neurological examinations were also performed every day, especially after each increase in traction length. If any complication occurred, such as upper-extremity numbness, the traction length was reduced to the previous length.

### Radiographic analysis

The major scoliosis and kyphosis (Cobb angle) were measured from biplanar, full-standing, standard radiographs of the whole spine at pre-traction, post-traction, and post-operative. Radiographs consisted of standing posteroanterior (PA) and lateral. In each case, radiographs were repeated every 2 weeks while the patients were in traction. Radiographic analysis of the coronal plane included the major curve Cobb angle on the standing PA radiograph. For the sagittal plane, we included the major curve Cobb angle and sagittal vertical axis (SVA), which was measured as the horizontal distance between the center of the C7 vertebral body and the posterior superior aspect of the S1 vertebral body. All measurements were performed using Surgimap software (version 2.3.2.1; New York, USA).

### Pulmonary Function Test (PFT)

The PFT was performed at both pre-traction and post-traction. The pulmonary function value included forced vital capacity (FVC), FVC %, forced expiratory volume at the end of the first second (FEV1), and FEV1%. To ensure accurate and reliable data, the PFT was measured in triplicate, and the highest values were selected. According to the American Thoracic Society’s guidelines for the severity of pulmonary impairment, ‘no’ pulmonary impairment was considered when the FVC% was > 80% of the predicted value, ‘mild’ when the FVC% was < 80% but > 65%, ‘moderate’ when the FVC% was < 65% but > 50%, and ‘severe’ when the FVC% was < 50% [8]. When the pulmonary function (in terms of the FVC %) at the completion of HPT had improved by at least 5%, the patients were defined as ‘pulmonary responders’ to HPT. If there was an improvement or decrease of 0–5%, patients were defined as ‘non-responders’ to HPT. If the FVC % dropped more than 5% during HPT, patients were defined as ‘decline’ [9].

### Statistical analysis

All of the data were analyzed using SPSS version 20.0 (SPSS, Chicago, IL, USA). Statistical data are presented as the mean ± standard deviation. Statistical comparison of the radiographic measurements and PFT results were performed using a parametric paired t-test; *p* < 0.05 was considered statistically significant.

## Results

### Demographics

**Fig. 2:**
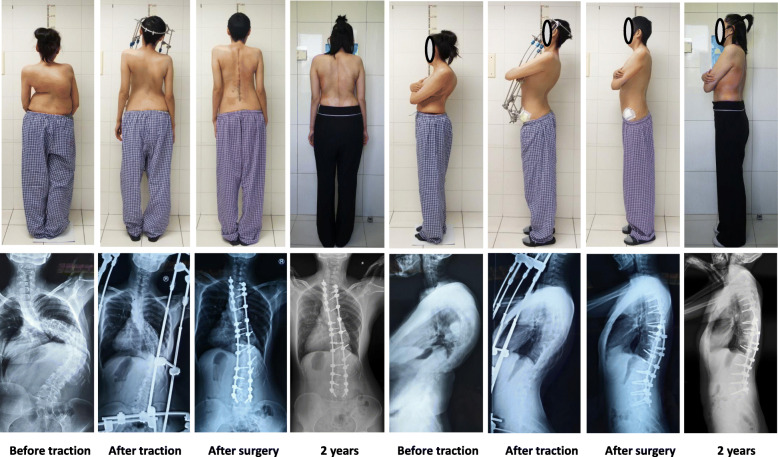
A 21-year-old female with congenital scoliosis and respiratory dysfunction. The major scoliosis was 120° and the major kyphosis angle was 74°before traction. After 5 weeks of HPT, the scoliosis and kyphosis had decreased to 63°and 46° respectively, and the FVC% had significantly increased from 36.00 to 57.57%. Posterior correction with instrumentation was performed. After surgery, the scoliosis had decreased to 45°, the kyphosis had decreased to 40°. At 2 years follow-up, no loss of correction was observed

**Fig. 3:**
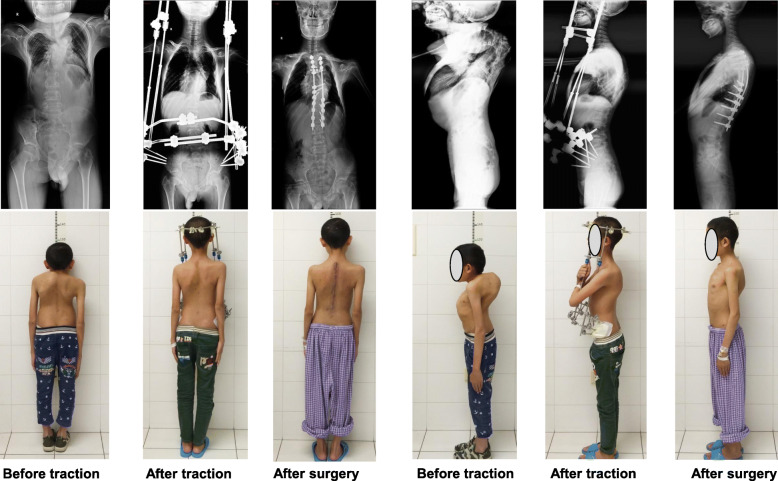
A 24-year-old female with severe kyphosis (165°) and respiratory dysfunction. After 6 weeks of HPT, the kyphosis had decreased to 55°, the scoliosis had decreased from 64° to 54°, and the FVC% had significantly increased from 34.10 to 44.69%. Posterior correction with Ponte osteotomy and instrumentation was performed. After the surgery, the kyphosis had decreased to 48°, the scoliosis had decreased to 42°

The study included a total of 30 patients undergoing HPT treatment, of which 22 were female (73%) and 8 were male (27%). The mean age of the patients was 30.00 ± 9.33 (12–46) years at the time of surgery. Etiological diagnoses were neuromuscular (*n* = 5), idiopathic (*n* = 15), and congenital (*n* = 10) scoliosis. All 30 patients were treated with posterior instrumentation and fusion without anterior release. Typical cases are shown in Figs. 2 and 3. The average pre-traction height was 139.63 ± 8.67 cm (124-160 cm), and the height after traction was 150.37 ± 9.08 cm (132-170 cm). The average increase was 10.73 ± 3.70 cm (6-15 cm; *p* < 0.001), and the average traction duration was 5.37 ± 0.93 weeks (4–6 weeks).

### Radiological parameters

**Fig. 4:**
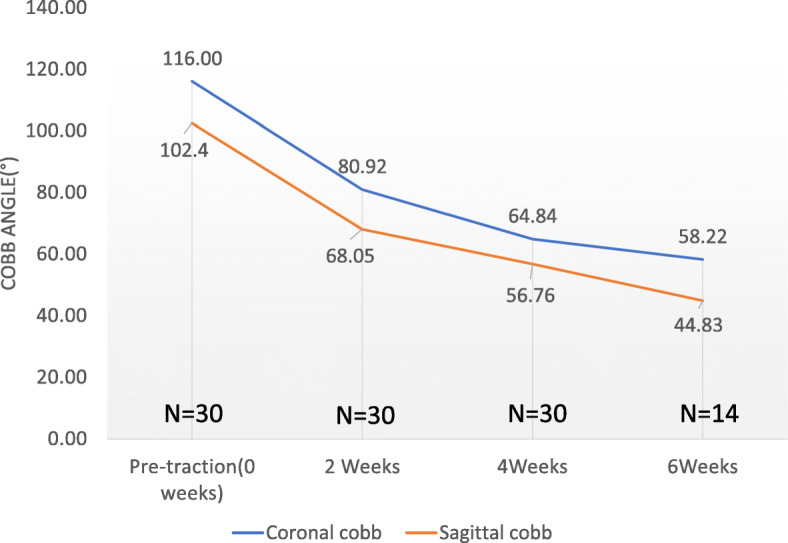
Improvement in coronal and sagittal plane deformities during the traction period (0–6 weeks, *n* = 30)

During traction, the major correction of kyphosis and scoliosis occurred in the first 2 weeks (Fig. 4). Over the first 2 weeks, correction of 30.24% for scoliosis and 33.54% for kyphosis was obtained. At 4 weeks, the correction of scoliosis and kyphosis was 44.10 and 44.57%, respectively. At 6 weeks, the correction of scoliosis and kyphosis was 49.81 and 56.22%, respectively. After HPT traction, the degree of scoliosis on the final-HPT radiographs demonstrated a significant improvement, as shown in Table 1. The major scoliosis correction averaged 52.77 ± 13.68° (4° to 75°; *p* < 0.0001) or 45.15 ± 11.41% (6–65%; *p* < 0001). After the surgery, the correction of scoliosis was 69.67 ± 13.11° (17°-94°; *p* < 0001) or 59.81 ± 8.82% (27–73%; *p* < 0001) compared with the curves prior to traction. Additionally, the major kyphosis presented a significant correction of 50.17 ± 22.81° (11–102°; *p* < 0.001) or 47.59 ± 13.33% (16–68%; *p* < 0.001) on the final-HPT radiographs and 60.33 ± 20.87° (24–105°; *p* < 0.001) or 53.67 ± 25.95% (23–69%; *p* < 0.001) after surgery compared with the kyphosis before traction. For the total correction, the HPT traction demonstrated an average correction of 74.46% for scoliosis and 81.23% for kyphosis.

**Table 1:** Radiographic parameters of the patients with severe scoliosis (*n* = 30)

	Pre-traction	Post-traction	Post-surgery
Scoliosis angle (°)	116.00 ± 16.70	63.23 ± 14.00^①^	46.33 ± 10.70^①②^
Kyphosis angle (°)	102.40 ± 27.67	52.23 ± 14.16^①^	42.0 ± 11.92^①②^
SVA (mm)	18.8 ± 36.3	−61.2 ± 35.6^①^	14.1 ± 23.7^②^

Note: ①Compared with pre-traction data, *p* < 0.05. ②Compared with post-traction data, *p* < 0.05

The pre-traction SVA was 18.8 ± 36.3 mm (− 35 mm – 97 mm). After HPT, the SVA was − 61.2 ± 35.6 mm (− 141 mm – 2 mm), and this demonstrated a significant change of − 80.0 ± 50.0 mm (− 7 mm – -178 mm; *p* < 0.001). The post-surgery SVA was significantly different relative to the post-traction SVA (− 75.3 ± 43.2 mm; *p* < 0.001) but similar to the pre-traction SVA (− 4.7 ± 35.4 mm; *p* = 0.475).

### Pulmonary function

The changes in FVC, FVC%, FEV1, and FEV1%, as shown in Table 2, were used to analyze the effect of HPT on pulmonary function. Regarding the results of the PFT, a significantly increased FVC was observed after HPT (0.91 L vs. 1.18 L; *p* < 0.001), with a significantly improved FVC% (34.15% vs. 44.18%; *p* < 0.001). Similarly, a significantly increased FEV1 was also observed (0.79 L vs. 1.02 L; *p* < 0.001), with a significantly improved FEV1% (34.10% vs. 44.25%; *p* < 0.001). At the time of pre-traction, 3 patients (10%) demonstrated moderate pulmonary impairment and 27 (90%) demonstrated severe pulmonary impairment. A total of 6 patients were identified as non-responders, presenting only a slight improvement in PFT, and the remaining 24 patients were classified as responders to HPT. The statistical analysis of the relationship between HPT and pulmonary function showed that there was no significant correlation between the improvements in the PFT results with the correction of the radiographic parameters (*p* > 0.05; Table 3).

**Table 2:** Comparison of PFT results before and after HPT (*n* = 30)

Parameters of PFT	Pre-traction	Post-traction	*p* value
FVC (L)	0.91 ± 0.42	1.18 ± 0.53	*p* < 0.001
FVC %	34.15 ± 13.98%	44.18 ± 16.26%	*p* < 0.001
FEV1 (L)	0.79 ± 0.41	1.02 ± 0.44	*p* < 0.001
FEV1%	34.10 ± 15.63%	44.25 ± 17.43%	*p* < 0.001

**Table 3:** Pearson’s coefficients between radiographic parameters and PFT results after traction (*n* = 30)

	△Major Cobb		△Kyphosis Cobb	
	Coefficient	*p* value	Coefficient	*p* value
△FVC	−0.253	0.052	0.158	0.224
△FVC %	−0.240	0.063	0.141	0.276
△FEV1	−0.234	0.076	0.211	0.104
△FEV1%	−0.245	0.193	0.160	0.218

△: The improvements in the PFT results. For example, △FVC means the improvement of FVC in post-traction compared with that in pre-traction

### Complications

Two patients developed complications after HPT traction. One patient experienced a decrease in muscle strength in the right lower extremity after 3 weeks of traction, and this patient recovered their muscle strength after the traction length was reduced to that of the previous length. Another patient experienced nausea and vomiting that resolved 1 week after halo placement, and this returned to normal after reducing the traction.

There was no pin loosening, pin site infection, nystagmus, paraplegia, cranial nerve injury, hypoglossal nerve injury, or any other kind of neurological complications observed. Because of the continuity of the traction, the patients often had cervical discomfort and trapezial soreness, and all these symptoms disappeared after undergoing surgery.

## Discussion

The rapid correction of severe scoliosis complicated with respiratory impairment can increase the risk of neurologic injury, morbidity, and mortality [10]. Halo traction has been used as an adjunctive method in the treatment of severe spinal deformities, which can reduce the risk of spinal cord injury while effectively obtaining correction of severe spinal deformities in a controlled and safe manner. As a result, less correction is needed during surgery, and aggressive procedures, such as vertebral column resection, might be avoided. Finally, this approach can also help improve pre-operative pulmonary function, allowing a better tolerance for more aggressive procedures [5, 6, 11]. Unlike HGT, HPT can provide powerful traction forces throughout the day to effectively correct spinal deformities. However, traditional HPT has a number of various complications and poor tolerance. As such, the use of HPT has gradually declined [12]. In this study, the pelvic ring of HPT device was a half-ring, and the rods were all placed on the anterolateral side of the truck (Fig. 1). This modified method allowed the patients to sleep in a supine position, wear clothes, and move by themselves while still achieving 24 h of continuous traction. The gradual traction applied during the pre-operative period might also help in evaluating the neurologic function and estimating the amount of correction that can safely be obtained.

O’Brien et al. first used HPT in the treatment of scoliosis in the 1970s [6], but there are few subsequent literature reports on the use of HPT for the treatment of severe spinal deformities, especially over the past 10 years. In this current study, with the help of HPT, the correction rate after HPT was 45.15% for scoliosis and 47.59% for kyphosis. This outcome was superior to the pre-operative HGT documented in prior reports, which demonstrated 15 to 38% correction in scoliosis and 17 to 35% correction in kyphosis [3–5, 11]. In the study by Janus et al., the correction of pre-operative HGT was similar to the final correction after surgery when compared with the initial curve (32% vs. 36% for scoliosis, 24% vs. 27% for kyphosis), but the total correction was significantly lower than that observed in this current study (36% vs. 59.81% for scoliosis, 27% vs. 53.67% for kyphosis) [13]. The correction of pre-operative HGT was similar to Janus in the studies by Koller et al. [9, 14]. Xia et al. reported a significant correction of spinal deformity conferred by pre-operative HGT in conjunction with posterior fusion; however, the correction conferred by pre-operative HGT in this prior study was lower (18.4 to 23%) [15], which confirms the effectiveness of our modified pre-operative HPT technique for improving severe spinal deformities prior to surgery due to the strong traction force and the subsequently reduced difficulty of the second-stage orthopedic corrective surgery. Moreover, the sagittal correction conferred by pre-operative HPT was superior to the coronal correction (45.15% for scoliosis, 47.59% for kyphosis), which was dissimilar to that reported for HGT [9, 13–15]. This difference might result because the force of HPT was primarily in front of the body in this study, or it might be due to the selection of patients with severe kyphosis. In this study, the sagittal imbalance of the patients became worse, with the center of the C7 vertebral body positioned further behind the sacrum after HPT traction. However, after surgery, the patients obtained sagittal balance. Thus, according to these results, the use of pre-operative HPT of severe spinal deformities had no measurable negative impact on sagittal balance.

Severe spine deformities can reduce the compliance of the respiratory system by affecting the thoracic cage as well as muscular and diaphragmatic function [16, 17]. When Cobb > 100°, respiratory system compliance is decreased to levels comparable to adult respiratory distress syndrome [8]. Therefore, halo traction is recommended to improve the poor respiratory function and to decrease complications in patients with severe spine deformities [9, 11, 18]. However, there are few reports regarding the effect of pre-operative HPT on the respiratory function in patients with severe spine deformities. In our sample, all of the patients had moderate or severe pulmonary impairment prior to traction, and significant improvements in the PFT results were achieved after HPT. These results indicated an increase of 10.03 ± 9.60% in the FVC%, which was similar to previously reported data on pediatric and adolescent populations. The mean change in FVC% after pre-operative HGT ranged from 10 to 14% [5, 9, 19]. This also confirmed that our pre-operative HPT could significantly improve pulmonary function in patients with severe spine deformities. However, it would still be necessary to use some pulmonary exercises to help improve muscle endurance and strength in these patients [11, 20]. We used feedback-breathing exercises in our patients to help improve endurance and muscle strength.

The length of traction depended on the response of the various curves to the traction and the patient’s systemic, respiratory, and nutritional conditions. Currently, the duration of HGT varies from 2 to 12 weeks, as based on reports in previous studies [5]. However, there is no consensus on the optimal duration of traction for HPT, and there are few studies reporting the relationship between the duration of traction and the correction of HPT. In this current study, the major correction of HPT on scoliosis and kyphosis was obtained in the first 2 weeks, and the correction plateaued at nearly 50% after approximately 4–6 weeks after a rapid period of initial correction. In regard to pulmonary function, Koller et al. reported that prolonged traction might not help further improve the pulmonary function [9]. For optimal correction and reduction in surgical risk, we recommend HPT traction for at least 4 weeks, and appropriately prolonged period of traction according to the patient’s condition. However, the optimal length of traction remains to be determined and requires additional studies.

**Fig. 5:**
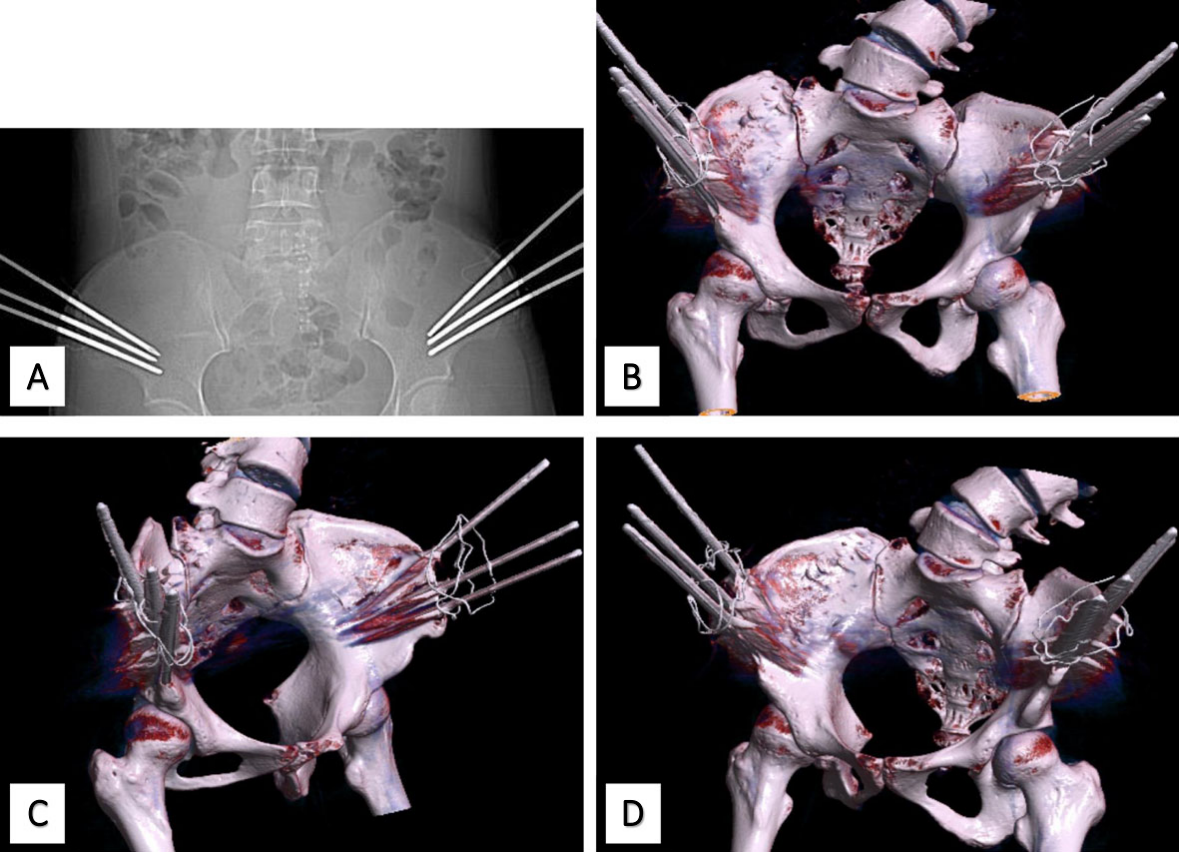
Photographs of bony pelvis with the pelvic pins. The anteroposterior digital radiographs (**a**), and the CT scan (**b-d**)

O’Brien et al. previously described the complications associated with HPT in detail. These included perforation of the intestine during insertion of the pelvic pins, infection of the pelvic pins, injury to the cervical spine, both acute and degenerative nerve injuries, and paraplegia [6]. Different from the HPT method used in the study by O’Brien et al., the pelvic pins used in the our HPT did not need to be drill through the iliac crest (Fig. 5), and there was no perforation of the intestine or injuries of other pelvic organs observed in this study. Strict protocols were used, including daily pin checks and prudent hygiene, and there were no other complications related to the pins, including infection of the pins or pin loosening. As for injury to the cervical spine, O’Brien et al. reported that more than 50% of patients showed some degeneration of the cervical spine when the traction was prolonged over 3 months [6]. Tredwell and O’Brien noted that the incidence of apophyseal joint degeneration was 47.4% in patients with HPT occurring over 9.47 months [21]. In this current study, the average time of traction was only 5.37 weeks, which was substantially shorter than that presented in O’Brien et al. or Tredwel and O’Brien. Additionally, significant degeneration of the cervical spine was not observed in our study; however, our patients often complained of cervical discomfort and trapezial soreness, but these symptoms disappeared after surgery. Neurologic complications, such as cranial nerve injuries and paraplegia, are known to occur during traction, and warning signs must be monitored daily. In our study, two patients (6.7%) developed neurologic complications after HPT traction, thought these occurrences returned to normal after reducing the traction. Moreover, due to the lack of immobilization of the patients receiving the modified HPT, any occurrence of osteoporosis could be avoided [22, 23].

To our knowledge, this was the first study to report on the use of this modified HPT, in which the pelvic ring was changed to a half-ring and the rods were all placed on the anterolateral side of the truck. All of these changes were made to improve patient tolerance and comfort while ensuring traction strength and effectiveness. However, this study has several limitations. The study was a retrospective review of a single cohort without comparison to a control group to confirm the efficacy of the HPT since the standard protocol in our center is surgical treatment after HPT in severe scoliosis cases. Secondly, this study is missing the post-operative data regarding PFT and the results of 1 year and 2 year follow-ups, as the acquisition of post-operative PFT was not a regular protocol and some patients did not achieve a 2 year follow-up. In the future, the results of 1 year and 2 years follow-ups will be assessed to complete the post-operative data set.

## Conclusions

This study indicated that the modified HPT could be used to help patients with severe spinal deformities complicated with respiratory dysfunction achieve significant correction in both the coronal and sagittal deformities during the pre-operative treatment period along with improved respiratory function and in the absence of severe complications.

## Data Availability

The datasets used or analysed during the current study are available from the corresponding author on reasonable request.

## Abbreviations

HPT: Halo-pelvic traction
HGT: Halo-gravity traction
SVA: Sagittal vertical axis
PFT: Pulmonary function test
FVC: Forced vital capacity
FEV1: Forced expiratory volume at the end of the first second
